# A comparison of how behavioral health organizations utilize training to prepare for health care reform

**DOI:** 10.1186/s13012-017-0549-0

**Published:** 2017-02-14

**Authors:** Victoria Stanhope, Mimi Choy-Brown, Stacey Barrenger, Jennifer Manuel, Micaela Mercado, Mary McKay, Steven C. Marcus

**Affiliations:** 1 Silver School of Social Work, New York University, 1 Washington Square North, New York, NY 10011 USA; 2 New York City Department of Health and Mental Hygiene, 42-09 28th street, Long Island City, NY 11101 USA; 3 Brown School of Social Work, Washington University, Campus Box 1196, One Brookings Drive, St. Louis, MO 63130-4899 USA; 4 School of Social Policy and Practice, University of Pennsylvania, 3535 Market Street, 3rd Floor, Philadelphia, PA 19104-2648 USA

**Keywords:** Research-practice partnerships, Policy reform, Organizational behavior

## Abstract

**Background:**

Under the Affordable Care Act, States have obtained Medicaid waivers to overhaul their behavioral health service systems to improve quality and reduce costs. Critical to implementation of broad service delivery reforms has been the preparation of organizations responsible for service delivery. This study focused on one large-scale initiative to overhaul its service system with the goal of improving service quality and reducing costs. The study examined the participation of behavioral health organizations in technical assistance efforts and the extent to which organizational factors related to their participation.

**Methods:**

This study matched two datasets to examine the organizational characteristics and training participation for 196 behavioral health organizations. Organizational characteristics were drawn from the Substance Abuse and Mental Health Services Administration National Mental Health Services Survey (N-MHSS). Training variables were drawn from the Clinical Technical Assistance Center’s master training database. Chi-square analyses and multivariate logistic regression models were used to examine the proportion of organizations that participated in training, the organizational characteristics (size, population served, service quality, infrastructure) that predicted participation in training, and for those who participated, the type (clinical or business) and intensity of training (webinar, learning collaborative, in-person) they received.

**Results:**

Overall 142 (72. 4%) of the sample participated in training. Organizations who pursued training were more likely to be large in size (*p* = .02), serve children in addition to adults (*p* < .01), provide child evidence-based practices (*p* = .01), and use computerized scheduling (*p* = .01). Of those trained, 95% participated in webinars, 64% participated in learning collaboratives and 35% participated in in-person trainings. More organizations participated in business trainings than clinical (63.8 vs. 59.2%). Organizations serving children had higher odds of participating in both clinical training (OR = 5.91, *p* < .01) and business training (OR = 4.24, *p* < .01) than those that did not serve children.

**Conclusions:**

The majority of organizations participated in trainings indicating desire for technical assistance to prepare for health care reform. Larger organizations and organizations serving children were more likely to participate potentially indicating increased interest in preparation. Over half participated in business trainings highlighting interest in learning to improve efficiency. Further understanding is needed to support organizational readiness for health care reform initiatives among behavioral health organizations.

## Background

The implementation of the Patient Protection and Affordable Care Act [1] has had a significant impact on the financing and delivery of mental health services. The expansion of health care coverage and the availability of financial incentives for system redesign have prompted states to restructure their service systems and develop standards to increase the accountability, efficiency, and quality of services [2]. A key part of implementing these large-scale state initiatives has been preparing individual organizations to adapt and thrive in this rapidly changing health care landscape [3, 4]. This study focuses on one such large-scale initiative enacted by New York State, which secured a Medicaid waiver to overhaul its service system with the goal of improving service quality and reducing costs. This transformation effort provided the opportunity to examine how individual organizations respond to broad state-level reforms in order to prepare for major shifts in service delivery.

Drawing from scholarship addressing the translation gap between research and practice, this study utilizes the strategy of a research-practice partnership to generate and disseminate knowledge related to the implementation of evidence-based practice (EBP) and policy. Partnership models emphasize both technical assistance and research. They provide the necessary training to practice and a natural laboratory for the generation of knowledge about EBP and translational efforts [5, 6]. As a result, these collaborations have the potential to rapidly deliver solutions for the pressing issues facing providers [5]. This study partnership was between university researchers and the Clinic Technical Assistance Center (CTAC) in New York State (NYS) [7].

Funded by the New York State Office of Mental Health in 2011, CTAC works in collaboration with service, advocacy, and technical assistance organizations to offer training, consultation, and educational resources to all adult- and child-serving mental health clinics in New York State (http://www.ctacny.org). Designed as an implementation strategy for New York State, one of CTAC’s goals is to facilitate the extensive changes in delivery and financing of behavioral health services required by their Medicaid redesign plan. New York State has recently acquired a Medicaid waiver in order to enact comprehensive delivery system reform aimed at improving service quality and lowering costs through the reduction of hospital admissions [8]. In this study, CTAC offered external technical assistance focusing on both the clinical and business needs of agencies to develop their capacity to deliver high-quality services within the context of new financial and regulatory health care reform directives. Providing this type of local technical assistance, which leverages the expertise of consultants who are familiar with local delivery systems is a recognized implementation strategy [9]. CTAC training materials and tools employ evidence-based approaches that reflect day-to-day clinical practice. Recognizing the need for different levels of training intensity and the reality of varying agency commitment, CTAC offers trainings via webinar, in-person, and intensive learning collaboratives (http://ctacny.org/our-offerings#).

A number of theoretical frameworks have been developed to understand the adoption and implementation of EBPs and quality improvement initiatives [10–14]. Common among these frameworks has been the influence of individual (clinicians, administrative staff), organizational, and community factors on implementation. Structural agency characteristics also have been important, including organizational size [15, 16] and funding sources [17]. For example, larger agencies have been associated with a greater likelihood of using EBPs compared to smaller agencies (e.g., [15, 16]), because they have greater resources, such as funding for training and supervision, to initiate changes in practice. The likelihood of implementing EBPs has also been associated with individual staff attitudes, knowledge, and experience [18, 19], climate and culture of the agency [18, 20], and infrastructure, such as physical space, staffing, and training opportunities [21, 22]. Together, these organizational practices, known as *institutionalization*, have facilitated not only implementation but also sustainability [23].

In addition to organizational characteristics, dissemination and implementation research has increasingly called for attention to the outer context, which includes social, policy, and financial environments [23], and a system’s perspective that takes into account the interrelationships among system elements and rules [24]. These two perspectives can either complement or contradict each other when implementing and sustaining new practices. Finally, concerns about scaling up and sustainability have expanded the focus of implementation research to go beyond adaptation of particular interventions to examine on a larger scale how practices are implemented in naturalistic environments [25]. Most research has examined scaling up and sustainability of a specific EBP [26], but more recently, studies have examined regional or state-based scaling up and sustainability of EBPs generally [2, 4, 27]. These studies have shown the importance of examining multiple sources of data at different levels of implementation to increase understanding of the complex processes associated with widespread adoption of multiple evidence-based practices within systems.

As there has been limited research on the adoption of large-scale state initiatives, CTAC has provided a valuable opportunity to better understand the uptake of evidence-based trainings and associated factors among behavioral health organizations. The purpose of this study was to examine the association between characteristics of behavioral health organizations (*N* = 196) in New York State and their participation in the technical assistance contracted through the NYS Office of Mental Health (OMH). The study aims were as follows: (1) to examine the rate of training participation among organizations, (2) to compare the organizational characteristics of those that participated in training to those who did not participate, and (3) to examine the type and intensity of training chosen by organizations and how their choices related to organizational characteristics.

## Methods

### Data sources

Data came from two sources which were matched according to organization. The first data source was the training participation records from the Community Technical Assistance Center (CTAC) and the second data set was the Substance Abuse and Mental Health Services Association sponsored (SAMHSA) 2008 National Mental Health Services Survey (N-MHSS) [28].

CTAC has offered training that is free-of-charge to all organizations with a licensed mental health clinic in New York State (*N* = 292). Clinics were notified about these trainings through emails sent by the Office of Mental Health, and subsequently through the CTAC listservs populated by online registrations. Records of the 187 trainings CTAC offered between November 2011 and March 2014, were utilized for this study. CTAC offered three types of trainings in clinical practices, business practices, and both practices (hybrid) at various intensities. The least intensive trainings were 1-h webinars. In-person trainings required full-day participation from agency staff. Learning collaboratives were the most intensive and required the greatest agency commitment, with regular group learning sessions and consultations over a 6- to 18-month period. Agency use of CTAC services and resources was voluntary. CTAC trainings have been described in detail elsewhere [29].

The CTAC database did not contain information about the characteristics of the participating organizations; therefore, we used the SAMHSA N-MHSS survey to look up information about their size, population served, service quality, and infrastructure. The N-MHSS is an annual survey that collects information about privately and publicly funded mental health treatment facilities in the USA. Facilities included in the survey were hospitals with psychiatric units, residential treatment centers, and outpatient facilities. Other sites of mental health service provision (e.g., correctional facilities, non-VA military facilities, or individual and small group practices) were excluded from the survey. Surveys were mailed and completed by facility directors. In 2008, 13,068 community mental health treatment facilities were surveyed with a response rate of 74% [28]. In New York State, 968 facilities responded to the 2008 survey from 330 organizations.

### Sample

**Fig. 1:**
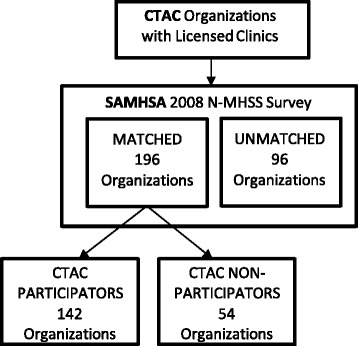
Data matching process

Data from both the SAMHSA N-MHSS and CTAC were aggregated to the organization level and matched on their organization name and address. Matching at the clinic level was not possible because this information was not available in the CTAC data. All organizations with licensed outpatient clinics in New York State (*N* = 292) serving children, adolescents, or adults were included in this study. Of these, 67% (*N* = 196) had matching SAMHSA data. No significant differences were found between matched (*N* = 196) and unmatched (*N* = 96) organizations on the key variables of organizational size and services provided (*p* = .266). Figure 1 describes the data matching process of New York State organizations with licensed clinics to SAMHSA N-MHSS.

### Measures

*Organizational Characteristics* were derived from the N-MHSS. *Organizational size* was measured using the number of facilities and size. Facility was measured according to whether or not an organization had a single facility, or two or more facilities. Size was defined as “large” for organizations with greater than 800 people receiving outpatient services or “small” for organizations with less than 800 people receiving outpatient services. *Population served* was defined based on whether the organization served children and adolescents and/or adults. *Service quality* was measured using variables indicating integrated, recovery-oriented care, and delivery of EBPs. Integrated care included organizations that reported providing chronic disease self-management services. Recovery-oriented care included organizations that reported consumer-run services. EBPs included organizations that reported using any of the following practices targeting children or adults (supported housing, supported employment, assertive community treatment, family psychoeducation, integrated dual disorders treatment, illness management and recovery, therapeutic foster care, multisystemic therapy, functional family therapy). *Infrastructure* was measured according to whether or not organizations used a computerized system for the following functions: test results reporting (e.g., laboratory results, psychological testing), treatment plan creation and maintenance, or patient scheduling.

Training participation, training type, and intensity of training were measured by variables from the CTAC database. *Training participation* was captured by whether an organization participated in CTAC trainings between November 2011 and March 2014. *Type* captured whether the training focused on *business practices* (i.e., Business Efficiencies and Effectiveness Project (BEEP), Business Effectiveness Assessment Module Practice Improvement Network (BEAM), or Change Action & Resource Exchange Network (CARE), *clinical practices* (i.e., clinical lunch and learn webinars, implementation of EBPs, practitioner education, and decision support); or *hybrid practices trainings*, which included both clinical and business content. *Intensity* was categorized as “low, mid, or high intensity” based on the type of modality training offered. One-hour webinar trainings were characterized as low intensity based on minimal time commitments required of participants. In-person trainings were characterized as mid-level intensity which required all-day time commitments by participants. Learning collaborative trainings were defined as high intensity and required participants to consistently participate in both in-person and web-based formats over several months.

### Analysis

Univariate analyses were conducted to analyze organizational characteristics and generate rates of specific types (i.e., any training, business, clinical, or hybrid) of training and associated confidence intervals. Chi-square analyses compared organizational characteristics of participators and non-participators. Among those who were trained, rates and confidence intervals for the percent were calculated for each training venue. Multivariate logistic regression was used to estimate the odds of training participation by organizational characteristics (i.e.., size, population served, quality services, and infrastructure). All analyses were conducted using SPSS.

Institutional review board approval was waived because the study was not considered to be a human subject research given that there was no interaction or intervention with individuals and no use of identifiable private information.

## Results

### Organizational characteristics

The overall matched sample was 196 organizations. For organizational size, the average number of facilities within each organization was 3.27 (SD = 3.759) with a range of 1–37. For population served, the average number of outpatient clients was 1,190 (SD = 1,299) with a range of 13–9,890. Of those organizations, 149 organizations served both children and adults, 10 served only children, and 37 served only adults. For quality of services provided, 59 (30%) delivered consumer-run services, 86 (44%) delivered chronic illness management practices, 85 (43%) delivered child EBPs, and 184 (94%) delivered adult EBPs. For infrastructure, 108 (55%) organizations used computerized results reporting, 137 (70%) organizations used computerized treatment plans, and 150 (77%) organizations used computerized patient scheduling.

### Training participation

Overall, 142 (72%) of the sample participated in CTAC training. Table 1 shows the characteristics of organizations that participated in the CTAC training versus those that did not participate. The two groups were significantly different with respect to organizational size, population served, delivering child EBPs, and utilizing computerized reporting and patient scheduling. Among the organizations who participated in the CTAC training, 67.5% had two or more facilities compared with only 50% in organizations who did not participate (*p* = .02). 54.8% of organizations that participated in trainings served more than 800 consumers as compared to 34.9% among those who did not participate (*p* = .02). 88% of organizations that participated in trainings served children as compared to 63% of organizations that did not participate (*p* < .01). 49% of organizations that participated in trainings provided child EBPs as compared to 28% of organizations that did not participate (*p* = .01). 50% of organizations that participated in trainings used computerized reporting as compared to 69% of those who did not participate (*p* = .01). 81.7% of organizations as compared to 63% of those that did not participate utilized computerized patient scheduling (*p* = .01).

**Table 1:** Characteristics of CTAC participators and non-participators

	Any CTAC participation (*N* = 142)	No CTAC participation (*N* = 54)	*P*
Size			
2 or more facilities	67.6% (96)	50.0% (27)	0.02
Large organizations (more than 800)	54.8% (74)	34.9% (15)	0.02
Population served			
Provide children services	88.0% (125)	63.0% (34)	<.01
Provide adult services	95.8% (136)	92.6% (50)	0.28
Service quality			
Consumer run services	31.7% (45)	25.9% (14)	0.27
Chronic disease/Illness management	45.1% (64)	40.7% (22)	0.35
Child EBP	49.3% (70)	27.8% (15)	0.01
Adult EBP	94.4% (134)	92.6% (50)	0.43
Infrastructure			
Computerized results reporting	50.0% (71)	68.5% (37)	0.01
Computerized treatment plans	70.4% (100)	68.5% (37)	0.46
Computerized patient scheduling	81.7% (116)	63.0% (34)	0.01

#### Training type and intensity

Table 2 shows the type and intensity of training in which the organizations participated. Business training was the most sought after type with 63.8% of organizations participating but a majority also participated in clinical trainings (59.2%). Of those organizations receiving any training, the largest majority engaged in webinars (95.1%), approximately two thirds (64.1%) engaged in learning collaboratives, and a third (34.5%) engaged in in-person training. Within each training type, webinars were the most utilized format with 88.8% of the business trainings, 100% of clinical training, and 76% of the hybrid trainings being webinar. However, within the organizations seeking business trainings, many also engaged in the learning collaborative format (62.4%).

**Table 2:** Training participation behavior by organizations

	Any training		Business		Clinical		Hybrid	
	*N* = 196		*N* = 196		*N* = 196		*N* = 196	
	%	CI	%	CI	%	CI	%	CI
Users of training	72.4	66.1–78.7	63.8	57.1–70.5	59.2	51.0–67.4	36.2	29.5–43.0
Training venues among those trained								
	*N* = 142		*N* = 125		*N* = 116		*N* = 71	
	%	CI	%	CI	%	CI	%	CI
Webinar	95.1	91.5–98.7	88.8	83.3–94.3	100	81.9–118.1	76.1	66.2–86.0
In-person	34.5	26.7–42.3	19.2	12.3–26.1	0	0	45.1	33.5–56.7
Learning collaborative	64.1	56.2–72.0	62.4	53.9–70.9	30.2	24.8–35.6	0	0

Table 3 shows that organizations providing children’s services were more likely to participate in trainings overall (OR = 2.73, *p* = .03) and more likely to participate in business (OR = 4.24, *p* < .01), clinical (OR = 5.91, *p* < .01), and hybrid (OR = 3.79, *p* = .02) trainings. Organizations with computerized results reporting were less likely (OR = .41, *p* = .04) to participate in training overall and less likely to participate in business (OR = .27, *p* < .01) and clinical (OR = .43, *p* = .03) training.

**Table 3:** Organizational characteristics predicting training participation (*N* = 196)

	Any participation				Business practices				Clinical practices				Hybrid			
	Rate (%)	P	AOR	P	Rate (%)	P	AOR	P	Rate (%)	P	AOR	P	Rate (%)	P	AOR	P
Population served																
Children services (*N* = 159)	78.6	<.01	2.73	0.03	69.8	<.01	4.24	<.01	66.2	<.01	5.91	<.01	40.9	<.01	3.79	0.02
No children services (*N* = 37)	45.9	–	–	–	37.8	–	–	–	24.6	–	–	–	16.2	-	–	–
Adult services (*N* = 186)	73.1	0.28	2.29	0.3	64.5	0.27	2.37	.30	57.4	0.14	2.76	0.2	36.6	0.48	1.62	0.54
No adult services	60.0	–	–	–	50.0	–	–	–	72.2	–	–	–	30.0	–	–	–
Size																
1 Facility (*N* = 73)	63.0	–	–	–	56.2	–	–	–	50.5	–	–	–	26.0	–	–	–
2+ Facilities (*N* = 123)	78.0	0.02	1.45	0.38	68.3	0.06	1.7	0.23	62.6	0.03	1.5	0.34	42.3	0.02	1.95	0.11
Large orgs >800 (*N* = 89)	83.1	0.02	1.48	0.54	69.7	0.21	0.97	0.95	56.2	0.06	1.41	0.38	43.8	0.1	1.26	0.53
Small orgs <800 (*N* = 89)	68.5	–	–	–	62.9	–	–	–	68.5	–	–	–	33.7	–	–	–
Service quality																
Consumer run (*N* = 59)	76.3	0.27	1.3	0.59	67.8	0.27	1.4	0.47	61.0	0.43	1.64	0.28	40.7	0.24	0.93	0.86
No Consumer run (*N* = 137)	70.8	–	–	–	62.0	–	–	–	58.4	–	–	–	34.3	–	–	–
Chronic disease management (*N* = 86)	74.4	0.35	1.44	0.39	64	0.54	1.28	0.56	53.5	0.1	0.67	0.33	41.9	0.1	2.03	0.07
No Chronic management (*N* = 110)	70.9	–	–	–	63.6	–	–	–	63.6	–	–	–	31.8	–	–	–
Child EBP (*N* = 85)	82.4	<.01	1.85	0.12	72.9	0.01	1.57	0.24	69.4	<.01	1.5	0.27	41.2	0.13	0.94	0.87
No child EBP (*N* = 111)	64.9	–	–	–	56.8	–	–	–	51.4	–	–	–	32.4	–	–	–
Adult EBP (*N* = 184)	72.8	0.43	0.55	0.48	64.1	0.45	0.48	0.45	58.7	0.41	0.37	0.3	35.9	0.45	0.54	0.44
No adult EBP (*N* = 12)	66.7	–	–	–	58.3	–	–	–	66.7	–	–	–	41.7	–	–	–
Infrastructure																
Results reporting (*N* = 108)	65.7	0.01	0.41	0.04	54.6	<.01	.27	<.01	50.9	<.01	0.43	0.03	32.4	0.24	0.54	0.1
No resultts reporting (*N* = 88)	80.7	–	–	–	75.0	–	–	–	69.3	–	–	–	40.9	–	–	–
Treatment plans (*N* = 137)	73	0.46	0.65	0.35	66.4	0.26	1.43	0.43	60.6	0.33	1	0.99	38.7	0.33	1.4	0.41
No treatment plans (*N* = 59)	71.2	–	–	–	57.6	–	–	–	55.9	–	–	–	30.5	–	–	–
Patient scheduling (*N* = 150)	77.3	>.01	1.54	0.39	70.0	<.01	2.23	0.1	62.7	0.05	0.91	0.85	38.0	0.39	0.75	0.54
No patient scheduling (*N* = 46)	56.5	–	–	–	43.5	–	–	–	47.8	–	–	–	30.4	–	–	–

## Discussion

Overall, the study found that the majority of behavioral health organizations participated in CTAC trainings. There were significant differences among organizations that chose to participate in the CTAC trainings and those that did not participate. Confirming prior findings, organizations with more facilities and a greater number of people served were more likely to participate in training. Higher participation rates among larger organizations could have been logistical, in that they have more staff who were interested in training, more infrastructure to support people who took time out from daily activities to be trained, and more resources to enact the service changes that might have been recommended as part of the training. Larger organizations may also have sought out training due to more leadership capacity to reflect and strategize about how best to position their organization for change. Referred to as *cosmopolitanism*, leaders of larger organizations often have had greater influence and access to policy makers, and therefore, more understanding about how policy changes have necessitated change in their organizations [11].

As the behavioral health community has braced for health care reform, there has been much anxiety about whether small organizations have the resources to survive changes in the rapidly changing landscape of mental health services delivery and financing [30]. Particularly, the need for considerable investment in infrastructure, such as health information technology data management systems, and the ability to negotiate partnerships with other health care providers would appear to favor larger organizations. A recent webinar by the National Council on Behavioral Health [31] answers the question “Why go big?” highlighting how size could increase organizational capacity to pursue value-based payment, community impact, and efficiencies in general. However, our study also found that organizations with computerized reporting were less likely to participate in trainings, maybe suggesting that organizations with existing infrastructure may have felt less need for technical assistance.

We found that organizations serving children and providing child EBPs were more likely to participate in the trainings. CTAC’s leadership has a specific expertise in child services and was founded focused primarily on organizations serving primarily children and families. Stronger relationships between child-serving organizations and CTAC may therefore explain higher rates of participation. Alternatively, health care reform for children’s services has lagged behind adult services potentially leaving these agencies with a more immediate need for training. The complexity of working with children and the greater involvement of other entities (e.g., the school system) may have also increased their training needs. Whatever the reason, organizations serving children were looking for more guidance on improving service quality and efficiency.

Among organizations who participated in training, the study demonstrated what type and intensity of training they preferred in order to prepare them for the new demands related to health care reform. Overall, organizations chose to participate in business training at a higher rate than clinical, which may be indicative of the nature of reform under the ACA. Still, a majority of organizations participated in clinical training. Although integrated care has demanded considerable improvements in terms of coordinated care and use of EBPs, the shifts related to business may have presented additional challenges for organizations. The ACA has used financial incentives that move away from fee-for-service models to reimbursement models that reward service quality over volume [32]. Integrated health care delivery mechanisms such as health homes have been tying reimbursement to outcomes, adding a new layer of complexity for organizations. Behavioral health organizations in New York have now been negotiating managed care contracts where they must use sophisticated business plans to demonstrate their ability to reduce costs and coordinate care with other provider organizations. This study found that organizations were more likely to use intensive learning collaboratives for business training than clinical training demonstrating a need for greater technical assistance in this area.

Overall, webinars were most utilized for clinical, business and hybrid training presumably due to the lesser burden on both the provider and organization. In a health services study implementing a quality improvement program, webinars and in-person trainings were found to have comparable results within a primary care physician sample, and webinars were identified to be more cost-effective and flexible to participant schedules [33]. Other research has found similar results supporting the preference of using webinar-based training to support clinical system changes such as electronic medical record implementation [34]. However, webinars are primarily didactic and have limited adaptability to particular participant needs. The more intensive learning collaborative format combined the format of webinars with in-person learning opportunities allowing for increased responsiveness to the individual participants’ needs. Given the speed of policy change, external technical assistance programs can provide efficient workforce development across organizations.

### Limitations

The study had a number of limitations. Because CTAC did not collect detailed clinic information in their attendance data, our analyses were aggregated to the organizational level. Conceptually, however, the implicit presumption was that clinic-level factors are representative of the broader organizational behavior and so our analyses at the organization level were appropriate and provided a significant opportunity to examine the relationships between organizational characteristics and training behavior. Still, data at the clinic level would provide a more fine-grained view of training behavior including possible variations across clinics within organizations. Second, our data did not collect information on whether organizations are participating in technical assistance/training support outside of CTAC and if that training was in addition to or instead of CTAC. Although this is a potential limitation, CTAC has been by far the largest provider of training to support behavioral health care reform in New York so it likely would have been considered as a primary source for webinars and in-person educational activities. Finally, the two data sources were collected at different time points with the organizational variables collected in 2008 and the CTAC data collected between 2011 and 2014. This may have limited our ability to capture the impact of current policy changes because during this time lag, there may have been developments in the structure and operation of the participating organizations. However, the factors we included in our analyses (organizational size and structure) have remained relatively stable and were not likely to have changed substantially over this time period.

## Conclusions

This study was able to demonstrate the interplay of outer setting factors driven by state-level policy changes and organizational factors and how they shape the uptake of health care reform. There has been a need to understand how organizations are responding to the need for training in new practices. Particularly important is to understand the organizational factors that enable organizations to adapt and succeed in this new climate of service delivery. This study confirms that size may be a key predictor of who seeks out training to help them succeed in this new environment. The potential for loss of smaller community-based behavioral health organizations presents a critical implication for the future of the behavioral health field. Further studies are needed to understand how organizations respond to broad policy changes and whether up-front investment in training leads to improved service quality and better fiscal outcomes. Also, as organizations utilize different training formats, more research is needed on the most effective and efficient types of training, particularly matching content to format. Specifically, qualitative research would enable us to discern the decision making processes underlying organizational training behavior. Research-provider partnerships, such as the one employed in this study, offer opportunities to further this type of research and build the evidence base in the complex area of large-scale implementation efforts.

## Abbreviations

ACA: Patient Protection and Affordable Care Act
BEAM: Business, Effectiveness Assessment Module Practice Improvement Network
BEEP: Business Efficiencies and Effectiveness Project
CARE: Change Action & Resource Exchange Network
CTAC: Clinic Technical Assistance Center
EBP: Evidence-based practice
N-MHSS: National Mental Health Services Survey
NYS: New York State
OMH: Office of Mental Health
SAMHSA: Substance Abuse and Mental Health Services Association
